# Researchers’ Perceptions of a Responsible Research Climate: A Multi Focus Group Study

**DOI:** 10.1007/s11948-020-00256-8

**Published:** 2020-08-10

**Authors:** Tamarinde Haven, H. Roeline Pasman, Guy Widdershoven, Lex Bouter, Joeri Tijdink

**Affiliations:** 1 Department of Philosophy, Vrije Universiteit Amsterdam, Amsterdam, The Netherlands; 2 Department of Public and Occupational Health, Vrije Universiteit Amsterdam, Amsterdam UMC, Amsterdam, The Netherlands; 3 Department of Ethics, Law and Humanities, Vrije Universiteit Amsterdam, Amsterdam UMC, Amsterdam, The Netherlands; 4 Department of Epidemiology and Data Science, Vrije Universiteit Amsterdam, Amsterdam UMC, Amsterdam, The Netherlands

**Keywords:** Research climate, Responsible conduct of research, Research integrity

## Abstract

The research climate plays a key role in fostering integrity in research. However, little is known about what constitutes a responsible research climate. We investigated academic researchers’ perceptions on this through focus group interviews. We recruited researchers from the Vrije Universiteit Amsterdam and the Amsterdam University Medical Center to participate in focus group discussions that consisted of researchers from similar academic ranks and disciplinary fields. We asked participants to reflect on the characteristics of a responsible research climate, the barriers they perceived and which interventions they thought fruitful to improve the research climate. Discussions were recorded and transcribed at verbatim. We used inductive content analysis to analyse the focus group transcripts. We conducted 12 focus groups with 61 researchers in total. We identified fair evaluation, openness, sufficient time, integrity, trust and freedom to be mentioned as important characteristics of a responsible research climate. Main perceived barriers were lack of support, unfair evaluation policies, normalization of overwork and insufficient supervision of early career researchers. Possible interventions suggested by the participants centered around improving support, discussing expectations and improving the quality of supervision. Some of the elements of a responsible research climate identified by participants are reflected in national and international codes of conduct, such as trust and openness. Although it may seem hard to change the research climate, we believe that the realisation that the research climate is suboptimal should provide the impetus for change informed by researchers’ experiences and opinions.

**Electronic supplementary material:**

The online version of this article (10.1007/s11948-020-00256-8) contains supplementary material, which is available to authorized users.

## Introduction

Breaches of research integrity have inspired studies into what drives researchers to engage in questionable research practices or research misconduct (Levelt Committee et al. 2012). Whereas initial explanations focused on the individual level, it has become increasingly apparent that the organizational research climate plays a key role in fostering integrity in research (Bouter 2015; Casadevall and Fang 2012; Sovacool 2008; Steneck 2002).

In this study, we define the organizational research climate as: “the shared meaning organisational members attach to the events, policies, practices and procedures they experience and the behaviours they see rewarded, supported, and expected.” (Schneider et al. 2013; Wells et al. 2014). Organisational culture, in contrast, can be defined as “the shared basic assumptions, values, and beliefs that characterise a setting…” (Schneider et al. 2013) (p. 362). In this paper, we therefore focus on the shared meaning researchers attach to the policies, practices and behaviours they associate with a responsible research climate, reasoning that it is easier to intervene on behaviour or policies, compared to intervening on values and beliefs.

Interest in researchers’ practices and behaviours in relation to research integrity can be traced back to Robert Merton’s (Merton and Storer 1973) scientific norms of disinterestedness, universalism, communality and organized skepticism and the emergence of the field of science and technology studies (STS). Later Zuckerman (Zuckerman 1977) built on Merton’s norms and connected a failure to uphold them to various forms of scientific fraud. For example, failure to uphold communality would lead to plagiarism. However, Merton’s norms have been criticised, with some questioning whether they are unique to science (Sismondo 2010), whether we need specific norms to describe scientific good practice (Schmaus 1983), and some contesting the idea of science as pursuing universal goals (Sismondo 2010).

Part of the critique came from researchers arguing that studying researchers’ behaviour should not be done by theorising using an outsiders’ perspective. Instead, to understand researchers’ behaviour, STS scholars argued that science had to be studied from within (Knorr Cetina 1995). These researchers, most notably Latour (1997), immersed themselves inside the laboratory to study first-hand the research climate as a phenomenon “defined by local rules and local knowledge” (Jasanoff et al. 1995) (p. 112). Recently, a Swiss research team investigated the local knowledge among Swiss biomedical scientists about research integrity, something they defined by referring to scientists’ personal responsibility to be honest and objective (Shaw and Satalkar 2018). Our research question is inspired by this bottom-up approach, specifically those rules related to a responsible research climate.

There is already some evidence, mostly quantitative, that the research climate can foster or undermine research integrity (Edwards and Roy 2017; Martinson et al. 2006; Seahsore Louis et al. 1995). For example, in a research climate where competition and suspicion among peers prevail, researchers seem to be more inclined to misbehave (Anderson et al. 2007; De Vries et al. 2006; Joynson and Leyser 2015). Conversely, in a research climate where new members were socialised into responsible research practices, researchers report less research misbehaviour (Anderson et al. 1994; Crain et al. 2013).

Existing codes of conduct for research integrity are aspirational when it comes to creating an environment conducive to research integrity, stating, for example, “Research institutions and organisations promote awareness and ensure a prevailing culture of research integrity.” (ALLEA 2017, p. 5) or “Institutions provide a working environment that promotes and safeguards good research practices.” (Netherlands Code of Conduct for Research Integrity 2018, p. 20). Yet it is not clear what this would look like in practice. With this study, we aim to explore researchers’ perception of a responsible research climate through focus group interviews. Specifically, we looked into three questions: (1) What are key characteristics of a responsible research climate? (2) What are the barriers to the creation of a responsible research climate? and (3) Which interventions alleviate barriers and improve the research climate where necessary?

## Methods

### Ethical Approval

All procedures performed in this study involving human participants were in accordance with the ethical standards of the institutional and/or national research committee (The Scientific and Ethical Review board of the Faculty of Behavioural and Movement Sciences (Vrije Universiteit Amsterdam), approval Number: VCWE-2017-017R1) and with the 1964 Helsinki declaration and its later amendments or comparable ethical standards.

### Participants

We included researchers that worked at the Vrije Universiteit Amsterdam and the Amsterdam University Medical Centre, location VUmc. The inclusion criterium was that the participants had to work in research for at least 1 day per week. Our recruitment strategy was threefold. We approached heads of department to ask for interested researchers, used our collegial network and randomly invited researchers by email to invite them to participate in our study. We aimed to recruit researchers for 4 discipline-specific focus groups (i.e. biomedicine, natural sciences, social sciences and the humanities) that were homogenous for 3 different academic ranks (PhD students, postdocs or assistant professors, and associate or full professors).

### Procedure

After confirmation of participation, participants received the information letter (Online resource 1) that included a link to our privacy policy (Online resource 2) as well as the informed consent form via email (Online resource 3). We sent these again one week prior to the focus group. The focus groups were conducted between March and May 2018. To ensure that participants felt safe to speak freely, we conducted the focus groups with researchers from similar ranks only. We conducted the focus group in English if there were participants that were not fluent in Dutch. We presumed that all researchers were proficient in verbalising themselves in English since English is considered the lingua franca of academia.

A moderator guided the focus group (JT or TH) discussion and an observer (internship student ES) made notes about the process and its content. The focus group started with a brief description of the project and its goals, as well as the goal of the focus group. Possible questions could be asked prior to signing the informed consent form.

We started the focus groups with a general task in which we asked participants to reflect on the responsible research climate by writing down three characteristics of a responsible research climate, discuss them with their neighbour and then share their insights with the group. We then put forth the question which barriers participants perceived for a responsible research climate and enquired which interventions could help to overcome these barriers. More detailed information can be found in our topic guide in Online resource 4. The focus groups took 90 min on average.

Within 10 days of the focus groups, we sent participants a short summary of the discussion and asked them for corrections to increase reliability (member check) (Meadows and Morse 2005). Participants agreed or provided minor suggestions that we incorporated prior to analyses. Recordings were transcribed by a transcription company under a data processing agreement. We used Atlas TI© Version 8.3.0. for the data analysis.

### Analysis

We used inductive content analysis to analyse the focus group transcripts. Inductive content analysis helps to bring down complex discussions to meaningful themes of interest (Elo and Kyngäs 2008). Two team members (TH and JT) analyzed and coded the transcripts independently. Individual analyses were then contrasted and discussed with two other team members (RP and GW) until we achieved consensus (Lincoln and Guba 1985).

Themes had to be relevant to our research questions. Specifically, we created separate coding schemes to visualize the characteristics of the responsible research climate (see Online resource 5). We did the same for the barriers for responsible research as well as interventions to improve the research climate where necessary (Online resource 6).

In our analyses, we focused on the research *climate* as defined in our introduction. We tried to code as openly as possible and looked for concrete behaviours, policies or practices related to each characteristic or theme. We acknowledged beforehand that the concrete behaviours that flow from a particular theme may be different for different disciplinary fields or academic ranks.

## Results

### Descriptive Information

We conducted 12 focus groups with 61 researchers across four different disciplinary fields and three academic ranks, see Table 1. In total, 36% was recruited through heads of departments, 7% through our own connections and the remaining 57% was randomly selected.

**Table 1:** Demographic information of focus groups participants

Academic rank	PhD student	Postdocs and assistant professor	Associate and full professor
*Disciplinary field*			
Biomedical sciences	5^/5^	5^/4^	4^/0^
Natural sciences	4^E/0^	3^/0^	4^/0^
Social sciences	4^E/3^	7^E/3^	4^E/1^
Humanities	6^E/5^	5^E/5^	7^/3^

^E^Focus group was conducted in English; other focus groups were conducted in Dutch

^/x^Number of female participants

### Responsible Research Climate

In the introduction of the focus groups, we asked participants to reflect on characteristics of a responsible research climate. Based on their discussions, we identified 6 characteristics that are presented in decreasing order of frequency: fair evaluation, openness, sufficient time, integrity, trust and freedom, respectively.

In what follows, we first describe the characteristic in general and how participants thought the characteristic could foster responsible research. We then elaborate on what sort of behaviors, policies or practices participants provided. Finally, we note whether the characteristic was described differently depending on participants’ academic rank or disciplinary field. Illustrative quotes per characteristic can be found in Table 2.

**Table 2:** Illustrative quotes regarding the characteristics of a responsible research climate

Characteristic	Quotation
Fair evaluation	I think an evaluation in which you can excel in one of the topics and don’t have to excel in all of them, so either you are required to have average scores on all topics that would be okay, or you can excel on a few of these and then perhaps not excel so much in others. I’m really fed up with all the boxes that have to be ticked and the list is getting longer and longer, and there’s no priorities there.—Assistant professor, social sciences I think more evaluations on a team base, so everybody has his own [strength], you have the one [colleague] that’s on the media, you have a colleague who’s writing grants and you have a colleague who’s writing more international journal papers or something like that.—Postdoctoral researcher, social sciences
Openness	…data sharing… allowing your data to be re-analyzed by other individuals to confirm the results that you have reported in your publication, and also allowing other researchers to incorporate those data into their own meta-analyses or allowing it to inform their research questions.—full professor, social sciences Openness is also that you feel open to discuss with others, if you feel that they are, maybe not mindfully, but they are doing things in a slightly different or wrong way in your opinion. That you can discuss this with the other person, without him or her feeling attacked by this. So that there is really an atmosphere of okay, we just trying all the best that we can and if somebody is doing something slightly wrong, it’s not a problem. We just work it out and we go on and we continue to do it better.—Associate professor, biomedical sciences
Sufficient time	The essence for conducting sound research is to have time and this time increasingly shrinking due to many disruptions and the loss of support staff—Full professor, natural sciences What is a research climate that I can work in responsibly without getting completely stressed out or anything? That means having time to think and write, because often the teaching time uses up all the research time so being able to protect that time.—Assistant professor, humanities And the second is time for research, we now have research time of less than a day a week and in that time, I can hardly read papers since in that time, I also need to supervise my students. When am I supposed to do my own research? I find that very unsatisfying. I enjoy teaching students, but I would like to define myself as a researcher, not primarily an administrator or a teacher—Associate professor, humanities
Integrity	Integrity is more something for yourself. So, you need to approach research with integrity and be the first to doubt your own research results—Full professor, natural sciences My point was mainly that there is an atmosphere where the professor gives the good example. But a good example is also truly listening, to people, to the data, to convey the attitude that the nothing but the truth matters—Associate professor, biomedical sciences
Trust	To trust both the one working beneath you as well those above you and to assume that they conduct good research and that they claim something for a reason—PhD student, biomedical sciences Scientific progress may look immense but it arises because we are with so many and all those baby steps eventually lead to giant leaps forward. And here reputation is of utmost importance, because you are in this international network in which you don’t always see what others have done in a different lab with their students, so if I try to repeat what a colleague from abroad has done and it does not work, then that trust is gone.—Assistant professor, natural sciences
Freedom	You need to have freedom in choice of topic… Ultimately you are best at judging what has potential in your area of expertise, what will lead to success or changes…—Full professor, natural sciences

### Fair Evaluation

Participants expressed that responsible conduct of research is partly fostered through fair evaluation of research and researchers’ performance. A fair evaluation was thought to be conducive to a responsible research climate as it could encourage researchers to perform responsible research and put less emphasis on citation criteria. In particular, this meant that research institutions should have sound policies about talent development, selection and promotion. Participants noted that there should be a formal evaluation system that is tailored to a researcher’s academic rank. More senior participants indicated that this meant assessing whether they mattered to the field and whether the papers they had written demonstrably had ‘impact’. The PhD candidate participants indicated that fair evaluation meant that their PhD thesis should not require three papers published in high-impact factor journals. Instead, evaluation of a PhD thesis should revolve around the quality of their research, independent of whether the results were positive or spectacular.

Relatedly, participants underlined that fair evaluation should include team-based performance evaluation and that teams need a diverse set of players. Responsible research evaluation then becomes more of a team endeavour. Participants in the biomedical sciences emphasised that studies are hardly ever done by one person. Participants from the humanities emphasised that team-based evaluation would allow them to appreciate that one colleague that is terrific at grant writing or that other colleague who is very skilled at writing international papers without perceiving them as competitors.

### Openness

Participants used the term openness as an umbrella concept that involved open research practices, open communication between colleagues and openness to collaborations.

Participants noted that openness about the conduct of research is a cornerstone of responsible research climate and involves conducting and reporting the research as openly as possible to transparently inform the reader about the results. Participants from the social and biomedical sciences mentioned multiple examples of openness that revolved around transparent ways of conducting research where ‘the research trail’ is traceable and verifiable. In addition, participants noted that openness included sharing data, methodology or codes where possible. Early career researchers said that there should be more openness in reporting as well. Early career researchers often felt as if important details were left out to prevent others from replicating the findings in question.

In addition, participants described that openness meant being open to colleagues in the department or research group. This included communication about expectations but also openness about mistakes that were made, so that these could be handled appropriately.

Besides, participants emphasized that in a responsible research climate, researchers are more open to collaboration. This form of openness could allow researchers to work with others that may broaden their professional horizon. Participants perceived that collaborations were often only initiated when there was some form of personal gain. In terms of behavior, openness to collaboration meant that researchers would actively seek out collaboration, especially interdisciplinary collaboration, to solve more complex problems as a collective.

### Sufficient Time

A dominant perception among focus group participants was that responsible research requires sufficient research time. To participants’ discontent, this research time was often overshadowed by other tasks (i.e. clinical, teaching or administrative duties). When time is short, research conduct could become ‘sloppy’. Participants acknowledged that good quality research takes time: time to keep up with developments in the field, to think, to read a paper, to produce a thorough manuscript or grant application review or to supervise students.

In addition, early career researchers emphasized that time is needed to make mistakes, learn and improve so that they can become responsible researchers. The participating senior researchers noted that they perceived difficulties prioritising their research, whereas in a responsible research climate, research is no hobby that one does on the side but one’s main focus.

### Integrity

Third, the topic of integrity pervaded many focus group discussions. In terms of behavior, integrity meant that researchers reflect critically on their own work. Furthermore, participants considered integrity to be conducive to a responsible research climate as it would involve department leaders encouraging their staff to develop their moral, and not just their scientific, competences. Lastly, to our participants, this includes having the right type of intrinsic motivation: the desire to do good research and to pursue the truth.

In addition, senior participants noted that there should be role models that conduct their work and supervision with integrity. This means there should be good examples, from starting with a good research question to being fair in scientific attributions and including all researchers that deserve authorship on the author list.

### Trust

According to the majority of the participants, trust is crucial in contemporary research with (inter)national and interdisciplinary collaborations. However, participants noted that trust is not sitting back and blindly relying on one’s peers. Instead, trust has to be sustained by actively holding each other accountable. One way to do this is through actively checking-in with peers and collaborators on how their work is going. This way, researchers can collectively hold expectations of good research practices in high regard. Senior researchers emphasized that in a responsible research climate, they should be able to trust those working below them to do their work with utmost care. Likewise, junior researchers stressed that they needed to trust that their supervisors know where the research projects are going.

### Freedom

Our participants indicated that freedom is vital for a responsible research climate. Regarding behavior, participants noted that there should be freedom to disagree with the existing scientific paradigm and to engage in scholarly debate. Freedom also meant that PhD students were encouraged to not passively accept their supervisor’s view, instead they should be encouraged to, when appropriate, challenge their supervisor’s view. When the research climate does not allow for this, false views may perpetuate for longer than needed. In addition, researchers from the humanities and natural sciences referred to the academic freedom to have autonomy in deciding which topics to study.

### Barriers

Participants described four main barriers to the creation of a responsible research climate: lack of support, unfair evaluation policies, normalization of overwork, and insufficient supervision. We describe them in order of frequency. Illustrative quotes per theme can be found in Table 3.

**Table 3:** Illustrative quotes about barriers to achieving a responsible research climate

Theme	Quotation
Lack of support	The teaching is pretty badly organized in general, so you have a lot of administrative burden aside from what you do with the students, which should be taken away, Assistant professor, social sciences Inconsequent technical support. Within our faculty, there has been a lot of changes in the technical support. For example, in our group, we had a technician that was the expert on all the lasers that we are using. And he was in the group for fifteen years and he knew everything about it. So, if any PhD was coming in new, they were trained by him. Then later on, they could do their own experiments. But at least this guy was always there and he was the person to train new people. And later on… there were less grants, so less people in the group eh, this person moved to another university.—PhD student, natural sciences
Unfair evaluation	A lot of it is dependent on the evaluation if you’re a good researcher, a particular evaluation of output across the different departments, trying to sort of find a scale that compares them all, which now has become the impact factor, is the holy grail. Impact factor based, so we, we align everybody along the same scale, the, regardless of sort of the history, the discipline, the, the nature, I guess the ease of publishing in the availability of journals and journal space etcetera eh, how, how you wanna see that. So that’s, that’s where you see that one thing has made it very difficult at the department level, is to manage your own policy eh, make your own decisions and, and make judgements on what you think is good and bad research and how you want to incentivize research…—Full professor, social sciences
Normalization of overwork	I once said to my supervisor that I had pain in my arms and neck from working. She said, yeah, I’ve had that for twenty years already so…—Postdoctoral researcher, social sciences The norm is that you are available full time, that is the norm. So, if you deviate from that [norm]… you don’t feel well or you think maybe I am going to get into trouble because I am not, now I am not responding—Assistant professor, humanities
Insufficient supervision	If you’re only trained by your promoter and your co-promoter, you get one world view about how you should publish things. And that’s not really the only world view that’s out there. And it’s then just up to your supervisor how flexible he is and his world view where he only accepts different approaches to research. So, I think in that way it can be really limiting to not have any experience with other research groups—PhD student, natural sciences There’s this huge amount of PhD students because they are cheap, and then the story is that you educate them and outside it would be helpful outside of academia, but from many PhDs perception I have the feeling that that’s not true.—Postdoctoral researcher I mean, now you can do a course to learn about teaching. But in the past, nobody learned how to teach. It was just something expected and based on tradition rather than the idea that you, well you might actually learn how to teach. So, I think this is the same with supervision… PhD student, humanities

First, participants perceived a lack of support from their research institution, which included bureaucracy. Participants reported an excessive administrative burden and talked about administrative systems that worked inefficiently. Some professors connected this to the university policy that had centralised all forms of support. In effect, this resulted in support staff that were generalists, who were often unable to help as promptly as the former department secretary could. Instead of simply delegating a task, professors sometimes spent half their day in requesting lab supplies whereas they felt that their time would be better spent on research.

Second, participants expressed concerns about unfair evaluation policies where the main focus was on publication quantity instead of on the quality of their scientific work. Related to this were concerns about the emphasis on impact factors, as one natural sciences professor put it: “It is not that I don’t want my paper to appear in Nature, but the reason that I would want it, is mainly due to the perception of policy makers or the university board, whereas there are other journals that are actually better, or at least, I would personally be happier for my paper to appear in one of those. You are pushed by people with little understanding of what you are doing, they now determine that you have to get published in Nature.” This discontent was mirrored in a discussion among biomedical sciences professors, who referred to this as the “impact fetish”.

In addition, participants in the humanities perceived that they had to excel in everything with little facilitation from their research institute in supporting them to develop professionally. Assistant professors said that they were on teaching positions without sufficient hours which meant that, in effect, teaching “ate up” their research time.

Third, participants talked about how their research climate normalized overwork and how everything had to go fast. Many described how it seemed to be expected of them to do the bulk of their research in their own time and how their working week often consisted of 60 h or more. Besides, participants felt stressed when they were not available as they noted their colleagues expected them to respond during the weekends and in the evenings. In other words: participants reported that it seemed the norm to be available all the time. Participants noted that this perpetuation of overwork was not conducive for a responsible research climate, as overworked researchers risk conducting their work less thoroughly than is desirable. Besides, some participants shared that they had experienced burnout symptoms, but that they were too afraid to lose their job and hence did not raise the issue.

Finally, participants noted that supervision of junior researchers often seemed suboptimal. PhD students described situations where colleagues were given 3 years of time to finish their thesis, but that their supervisor expected the quality of a four-year thesis. Besides, PhD students were aware that the odds of a future academic career were low, but reported that their supervisors devoted no attention to a possible career outside academia. Senior participants expressed concerns about the lack of role models who convey responsible research, whereas more junior participants underlined how to do research is mostly conveyed by the supervisor. Both PhD students and senior researchers noted that there was little guidance on how to become a good supervisor or a role model that fosters a responsible research climate.

### Interventions

Finally, participants discussed several interventions to overcome the beforementioned barriers to a responsible research climate. We identified three themes related to interventions that participants thought to be conducive to a responsible research climate: improve researchers’ support, discuss expectations, and improve quality of supervision. Illustrative quotes per theme can be found in Table 4.

**Table 4:** Illustrative quotes about interventions to improve the research climate

Theme	Quotation
Improve support	In terms of say, the respect for professors to make decisions versus the authority to make [hiring] decisions… And it’s a really negative signal of trust, if you are not being seen as the one who can actually best think about hiring decisions, about promotion decisions, about what task you want to whom.—Full professor, natural sciences
Discuss expectations	I’m aware of colleagues who are very conscious about when they are sending emails. Opposite to what you’re saying, they work on the weekends but they make sure not to send their PhDs replies on weekends or in the evenings because they don’t want to get that message across.—Assistant professor, social sciences
Improve supervision	PhD student 1: there are courses for principal investigators on how to supervise PhD students but they all don’t have time… PhD student 2: or you should make it compulsory, that they have to repeat the course each year or something… PhD student 3: yes, and if you don’t pass, you are not allowed to be a supervisor!—PhD students, biomedical sciences For example, I once took a course about supervising PhD students. Well, at that point the part on integrity was really small, it has increased somewhat in recent years. But there is a natural role for good supervision in your training as a researcher or clinical professional.—Associate professor, biomedical sciences

### Improve Support of Researchers

First, participants mentioned that researchers could be supported in different ways: by decreasing the administrative burden, by sound research evaluation policies and by creating formal research time. For example, participants emphasized the need to diminish administrative hassles by investing in support staff. They noted that support staff hours should be included in grant applications.

Additionally, participants discussed the creation of evidence-based research evaluation policies that acknowledge scientific excellence in different ways. Concretely, participants noted that funding volume and number of publications should not be the sole criteria for promotion. Instead, professors and group leaders should be consulted regarding whom they thought suitable and why. Additionally, other criteria such as public outreach, outstanding teaching qualities and cross-disciplinary collaborations should also be considered. Lastly, participants explored the idea of team-based evaluations so that individual researchers need not excel at everything on their own.

Besides, participants discussed creating formal research time. This included constructing periods in the academic year where researchers had no teaching duties and their administrative duties were kept at a minimum. For clinicians, this meant their supervisors needed to support them in protecting their research time whilst specializing. Participants stressed that this required a change in attitude among researchers: instead of treating research “as a hobby” that you do on the side, researchers needed to take pride in their research time.

### Discuss Expectations

Also, participants noted that it should be more accepted to set limits about what to do. Participants contended that it is healthy to do something else than work. They noted that in order to change the current existing climate, team members should be transparent and openly discuss expectations regarding (un)availability. Participants stressed that this discussion needed to involve PhD students. Relatedly, PhD supervisors had to be aware about the (unintended) expectations they might convey when sending PhD students emails in the evening or over the weekend.

### Improve Quality of Supervision

Lastly, participants underlined that to improve the quality of supervision, it should be formalized what is expected of a PhD supervisor. Participants in the biomedical sciences coined the idea of creating a discipline-specific manual listing supervising duties. Other participants mentioned peer support groups for supervisors where they could discuss supervision-related dilemmas with their peers. In addition, participants emphasized that there should be training modules available for PhD supervisors that focus on cultivating responsible role model behaviors.

## Discussion

This focus group study investigated academic researchers’ perceptions of a responsible research climate, which barriers these researchers perceived in fostering a responsible research climate, and which interventions they considered beneficial for improving the research climate when necessary. In what follows, we reflect on our findings, connect them to existing literature, briefly consider the differences between academic ranks and disciplinary fields, and discuss the strengths and weaknesses of our study.

### Connection with Research Integrity

It is important that the barriers we describe are not necessarily leading to an irresponsible research climate. They are interconnected factors that may hamper research integrity in various (and often indirect) ways, below we critically examine the barriers our participants discussed.

Take normalisation of overwork, there are different ways in which this could hamper the development of a responsible research climate, but it need not do so per se. To our participants, a systematic state of overwork (or even burnout, see Boyd 2014) could increase the chance that researchers engage in sloppy science (unintentionally, but in an overworked state, a researcher is less likely to notice errors, inconsistencies or flaws) or give in to temptation (when a researcher is overworked and frustrated, she may be more likely to incorrectly round off the obtained *p*-value).

Another example would be unfair evaluation, it is almost never the case that a system is unfair to everyone, rather it is unfair because it favours some over others. Hence researchers working on eye-catching topics may thrive in an evaluation system based on impact factors. But for many of our participants, it hampered a responsible research climate because the “impact fetish” steered researchers away from supervising or peer review, research-related activities that are also important (Moher et al. 2020).

Relatedly, readers may have experienced a lack of support somewhere in their academic career, but was this associated with an irresponsible research climate? To some of our participants, it was a form of research waste: valuable grant money is put towards a professor’s salary with the idea that he or she uses that time to coordinate a study, not with the idea that the professor’s days are spent on ensuring lab supplies. For junior researchers who were not charged with running a lab, the lack of support meant that they struggled to learn the right skills required to do their research, as the lab support staff who were extremely well-versed in complicated lab techniques got laid-off. For those outside a lab, the lack of support meant that it was hard to find sufficient time to sit down and write a sound paper. According to them, academic writing is not something one can do in between various administrative tasks, it requires time to engage with a topic, find the right words to succinctly convey the findings and integrate different perspectives. The idea here would be that if senior staff are forced to spend rather large stretches of their day on administrative tasks, this may negatively impact the quality, rigor and thereby integrity of their work.

Finally, many PhD students may, in hindsight or currently, describe their supervision as suboptimal. Can this not just be interpreted as PhD students complaining about their superiors, as employees in other work environments will do from time to time? It is our understanding that participants recognised the challenges of good mentorship and that they saw a clear role for the supervisor in conveying responsible conduct of research through role modelling and responsible supervision (Bird 2001). This is not to say that without a responsible role model, PhD students would go astray. Yet, with an irresponsible role model, it may be more likely that PhD students internalise flawed research practices.

### Responsible Research Climates

The term, responsible research climate may remind readers of a field called Responsible Research and Innovation (RRI) that has gained attention thanks to the European Commission’s emphasis on the topic in its Horizon 2020 funding calls. Where RRI focuses on public engagement and societal relevance of research, RCR concerns behaviours that influence the validity of, and the trust in, research (World Conferences on Research-Integrity 2020). Nevertheless, some studies that looked into factors that hindered or facilitated RRI can be illuminating for RCR as these studies looked at the research climate as well.

A case study of what RRI looks like in practice that was conducted at two Dutch research universities (Wageningen University and Radboud University Nijmegen) around the same time as our study allows us to compare and contrast our results (Van der Molen et al. 2018). One of the main barriers to a fruitful uptake of RRI that their interviewees reported was the mismatch between researchers’ wishes (e.g. to conduct research that is relevant for society) and the way in which they were formally evaluated and rewarded that emphasised publications and grants (Van der Molen et al. 2018). This resembles what our participants described as unfair evaluation, although our participants did only occasionally mention societal relevance.

The RRI case study listed the “autonomy-oriented academic culture” (p. 58) at Radboud as a barrier for implementing compulsory research integrity training for senior researchers. This could be interpreted as a clash between two of our characteristics of a responsible research climate: freedom on the one hand [especially among “anti-hierarchical” senior researchers (p. 58)] versus integrity on the other hand. What this example illustrates is that creating a responsible research climate requires great care as predominantly focusing on one characteristic may come at the expense of another.

### Comparison with Existing Literature

The characteristics that our participants associated with a responsible research climate may not surprise the reader. Here we compare and contrast our findings with existing literature, in an attempt to show that at times, new or different perspectives can be revealed.

Openness among colleagues and openness to collaboration have previously been mentioned as conducive to responsible research. Based on a survey and focus groups among U.K. academics, Joynson and Leyser reported openness and collaboration to be pivotal for high quality research (Joynson and Leyser 2015).

Related, Munafò and colleagues reported a variety of ways to make research more open and thereby more responsible (Munafò et al. 2017). In line with the goals of open science, they encouraged open data, open software and open materials, which was something our participants discussed too. However, there are also differences: Munafò et al. (2017) also elaborate on the benefits of initiatives such as registered reports, preprints or preregistration, whereas these initiatives were not mentioned in our focus group discussions. This indicates that certain open science related initiatives may be more accepted by the scientific community overall than others, perhaps because open data or open software are more broadly considered to be relevant compared to, for example, preregistration.

Finally, scholars that study the organisational climate have consistently emphasised the importance of fairness of internal processes, such as promotion and evaluation (Gorsira et al. 2018; Schneider et al. 2013). It is thought that working in a climate where one is treated fairly, one is more likely to abide by organisational rules and procedures. Applied to the research climate: when researchers feel fairly evaluated, they may be less likely to cut corners (Martinson et al. 2010).

Finally, improving supervision has previously been discussed as something that institutions should provide clearer guidelines for (Kornfeld 2012). Our participants extended this with concrete examples of how to formalize what is expected of a supervisor by means of a supervision manual or through training programs that focus on responsible supervision. Guidance on good mentoring is not uncommon in The Netherlands, as indicated by a qualitative study amongst PhD students and PhD supervisors by Maastricht University (Van der Boom et al. 2013; Woolderink et al. 2015). They recommend making expectations mutually explicit and emphasized clear and constructive feedback. Furthermore, Leiden University has a best practices manual for supervision (Leiden/University, https://www.universiteitleiden.nl/binaries/content/assets/geesteswetenschappen/pdfs/best-practices-for-phd-supervision.pdf) where they translate commitments into concrete actions that both the PhD supervisor and the PhD student can take. One possible avenue would be to incorporate good mentorship into the reward system, making it a scientific activity that is valued in its own right, as described in the recently released Hong Kong Principles for Assessing Researchers (Moher et al. 2020). Principle 5 reads: “Value a range of other contributions to responsible research and scholarly activity, such as peer review for grants and publications, mentoring, outreach, and knowledge exchange.” (p. 11). These contributions could be incorporated into the talent development policies that our participants discussed, but their possible effects on the research climate should be examined with due scrutiny.

Future studies examining these intervention effects (here: incorporating supervision into the evaluation criteria) should be explicit about where it is they expect change to occur. For example, would they expect a change in the perceptions of the research climate among PhD students or a decrease in dropout among PhD students? It is beyond the scope of this study to define a precise way to measure this or any other effect. We also note that evaluating the effects of interventions to promote RCR can be challenging, a Cochrane review of 31 educational intervention studies to promote RCR found hardly any effect (Marusic et al. 2013).

### Differences Between Disciplinary Fields and Academic Ranks

In our analyses, we focused on broad characteristics and we did occasionally find differences in the way in which a particular characteristic was operationalised. When reviewing the possible differences between academic ranks, the idea of teaching-free periods pervaded discussions among postdocs and assistant professors and was less pronounced among focus groups with PhD students, possibly because not all PhD students have formal teaching duties. Similarly, some participating biomedical researchers noted that it was not teaching that "ate up" their research time, it was that their clinical duties were prioritised regardless. Yet, these biomedical researchers too contended that good research practices benefit from sufficient time. All in all, we take these differences to be a matter of degree and believe that the characteristics we identified could bear relevance for researchers across academic ranks and disciplinary fields.

### Strengths

This is the first study to systematically investigate perceptions of a responsible research climate across academic ranks and disciplinary fields (that is, across the biomedical sciences, natural sciences, social sciences and the humanities). Most of the published focus group studies about good research practices thus far focused on a particular disciplinary field, e.g. social or biomedical science (Anderson et al. 2007; De Vries et al. 2006; Tijdink et al. 2016). Because our sample included a diversity of academic ranks and disciplinary fields, we hope that our results are relevant for researchers regardless of their specific academic rank or disciplinary field.

We believe we were able to unpack what constitutes a responsible research climate by tangibly describing how participants characterised a responsible research climate. This, together with the interventions that were brought up by our participants, can provide a start for an evidence-based debate about fostering a responsible research climate.

### Weaknesses

In light of our results, there are some limitations that need to be addressed. First, we aimed to recruit more participants and especially more female participants, particularly among the more senior ranks and in the natural sciences. This unequal composition reflects a national finding: Whereas Dutch universities have taken measures to increase the percentage of women in higher ranks of academia, the percentage of women gets lower with higher functions. We therefore acknowledge the possibility that female-specific barriers were overshadowed or that some barriers were more pronounced for female researchers than we could find out due to our focus groups’ gender composition.

Second, this study did not include participants from the technical and engineering sciences. That leaves the option open that we have missed key characteristics of responsible research for technical scientists and engineers. However, this is a sample-specific deficit since Amsterdam does not have technical or engineering science faculties.

Lastly, we focused mainly on behaviours, policies and practices which provide tangible results but may oversimplify certain issues. In addition, focusing on these behaviours does not allow one to infer the drivers of those same behaviours based on the same data. All in all, our data regard *which* characteristics researchers associate with a responsible research climate but not *why* they associate certain characteristics with a responsible research climate.

## Conclusion

It may be hard to change the research climate. Alike the meteorological climate, an analogy that we borrow from the creators of the SOuRCe^©^ (Martinson et al. 2016), the research climate is influenced by different factors, such as individual researchers and the system governing academic science. But to stick to this analogy, it is the realisation that there is something wrong with the climate that can spark behavioural change. The interventions that we listed could give that behavioural change concrete shape. We hope that future research will explore their feasibility and effectiveness.

## Electronic supplementary material

Below is the link to the electronic supplementary material.

1. Information letter: Information letter for participation in the focus groups (DOCX 17 kb)

2. Privacy policy: Privacy policy for participation in the focus groups (DOCX 31 kb)

3. Informed consent form: Informed consent form for participation in the focus groups (DOCX 16 kb)

4. Topic guide: Guide that addressed the questions posed in the focus groups (DOCX 19 kb)

5. Coding scheme characteristics (PDF 233 kb)

6. Coding scheme barriers and interventions (PDF 240 kb)
