# Supplementary material for: Researchers’ Perceptions of a Responsible Research Climate: A Multi Focus Group Study
Source: Sci Eng Ethics. 2020 Aug 10;26(6):3017–36. doi: 10.1007/s11948-020-00256-8 (PMC7755866; doi:10.1007/s11948-020-00256-8)
Supplement: Supplementary file 1 — 1. Information letter: Information letter for participation in the focus groups (DOCX 17 kb) [file 11948_2020_256_MOESM1_ESM.docx]

*Information letter focus group Academic Research Climate Amsterdam (ARCA)*

Dear colleague,

With this letter, we want to give you some more information about your participation in the ARCA focus group. The focus group will be held on date XXXX in room XXX and will take approximately 90 minutes. This letter contains information about the importance and aim of the study, the rules of the focus group and some info on how the data is processed.
 **Importance**
Responsible conduct of research is pivotal as a scientist. The ARCA project investigates the academic research climate and we use focus groups to explore what factors hinder or promote responsible research. With this knowledge, we can better inform policy makers about a healthy and responsible research climate.

**Aim**The aim of the focus groups is to get a better grasp of an optimal research climate within various disciplines. With the help of a skilled moderator, various themes and topics are discussed in relation to the research culture. Aside from that, we want to know to what sort of barriers researchers may experience for responsible conduct of research. With this information, we want to get more insight into what sort of interventions would be fruitful to improve the research climate where necessary.

**Focus group**
We ask you to take part in our focus group and to share with us your knowledge, experience and insight as a researcher together with other researchers from your field. Your participation and everything that is discussed during the focus group is strictly confidential.

We (the study’s researchers) analyse the transcript of the focus group discussion confidentially (and anonymously where possible) to identify recurring themes. After participation, you will also receive a short summary of the things discussed and will be asked to review our reporting.

**Data processing**
The data collected in the focus groups will be analysed confidentially for recurring and important themes. Also, there will be some quotes highlighted to illustrate the themes discussed. These occur, stripped of personal information, in a scientific publication. More elaborate information can be found in our [link privacy policy].

If you have any questions upon reading this letter, feel free to reach out to me, [name researcher], via [address researcher]

Kind regards, on behalf of the research team,

[Name researcher]

[website research project]
