# Supplementary material for: Researchers’ Perceptions of a Responsible Research Climate: A Multi Focus Group Study
Source: Sci Eng Ethics. 2020 Aug 10;26(6):3017–36. doi: 10.1007/s11948-020-00256-8 (PMC7755866; doi:10.1007/s11948-020-00256-8)
Supplement: Supplementary file 2 — 2. Privacy policy: Privacy policy for participation in the focus groups (DOCX 31 kb) [file 11948_2020_256_MOESM2_ESM.docx]

**Privacy Policy**

Project Academic Research Climate in Amsterdam (ARCA)

**Introduction**

This privacy policy informs participants how we comply with the privacy regulations of the participating institutions. We have received your email address from the Executive board of one of the four academic institutions in Amsterdam (VU, VU University Medical Center, University of Amsterdam and Academic Medical Center). With the boards of these institutions, we have made a data transfer agreement to obtain the email addresses of all active scientists. In this agreement, we are committed to the privacy laws of the four institutions. The agreement stipulates that we will code all data after sampling and anonymize all obtained data before analysis. The institutions will have no access to the research data. They will only be informed with aggregated reports on a faculty level as specified below.

For the focus groups, the participating institutions will receive no reports. The results of the analysis of the anonymized transcripts of the focus groups will be published in a manuscript for research purposes. Finally, the two interventions intended to improve the research climate are advertised throughout the institution, but all data learned from participant’s experiences will be anonymized.

**Aim of the study**

The goal of the ARCA project is to map the research climate within the four academic institutions in Amsterdam.

**Ethical considerations**

The ARCA processes, manages and secures personal information with the utmost care. We work according to the [Dutch Code of Conduct for Scientific Practice.](http://www.vsnu.nl/files/documenten/Domeinen/Onderzoek/The_Netherlands_Code_of_Conduct_for_Scientific_Practice_2012.pdf)

Therewith we comply with all the requirements of the Dutch Personal Data Protection Act. The study protocols were reviewed by the Medical Ethics Committee (METc) of the VU University Medical Center. The METc reviewed the protocol and stated that the study does not fall under the scope of the Medical Research Involving Human Subjects Act (WMO). The study protocol was reviewed and has been approved by the Ethics committee of the VU, faculty of Behavioral and Movement Sciences.

**Scope of this Privacy Policy**

This privacy policy applies to all personal information we collect as part of the ARCA project. This regards three types of data: (1) a database of the responses to a survey questionnaire sent out via e-mail and (2) verbal responses during focus groups where we will ask invitees to share their views on the research climate and (3) responses as to how people experienced our pilot training and moral case deliberations (reviews/ survey responses).

1. Responses to the survey questionnaire

After survey data collection, we will de-couple email address from responses after which email addresses will be deleted from the database before analysis and aggregation of data. Only data academic rank, gender and disciplinary field (fields are categorized into four types: life and medical sciences, natural and engineering sciences, social and behavioural sciences and finally humanities, language, communication law and arts) will be used. These procedures described here will effectively anonymize the data to assure that it will be practically impossible to trace back personal responses to specific questions.

1. Focus group data

For focus group data, we will provide all potential participants with an information letter and ask informed consent beforehand and do a thorough verbal instruction at the start of the focus groups. The third party that will transcribe the focus groups will sign a confidentiality form and will be asked to transcribe and recode respondent’s names into unidentifiable respondent’s codes. Specific quotes from the focus groups that could appear in publications should not be recognizable by anyone who did not attend that specific focus group. Quotes will only be published in an effectively anonymized fashion (e.g. Respondent 1: “insert quote…”).

1. Interventions

For the first intervention, a pilot training for novice PhD supervisors, we will be sent out a brief questionnaire after each training day to all participants. Here we will confidentially ask about their experiences, what they liked about the training and what they missed. At the final day of the training, we will also ask the participants to verbally give comments and tips for us how to optimize, promote and expand the training. This data will be effectively anonymized before publishing and will only be used to improve and promote the training.

For the second intervention, the moral case deliberations, we will ask all participants whether they are okay with the global themes of the session to be recorded. The exact dilemmas discussed will never be available. We will record the time and date of the moral case deliberation, as well as some characteristics of its participants (only their rank and disciplinary field) to assess the diversity of the participants. Furthermore, we will ask participants to share their experience with the moral case deliberation technique, how they perceive this technique for research integrity and the specific session they participated in. These responses will be gathered confidentially and anonymously.

**Provision of anonymized data to third parties**

Specifically for the survey:

The de-identified data (raw data after removal of email address) will not be provided to third parties unless:

- This is necessary for the purpose for which the data were obtained (i.e. submission of the de- identified dataset as part of publication policies).

- When our anonymized data is needed for research purposes in the future (i.e. a collaboration with another institution), your identity will remain protected and data will only be available after a data transfer agreement is signed by the requesting party and the researchers.

- When such a request for the anonymized data is issued, data will only be shared if the main research team reaches consensus that the requester’s use of the data for future research is strictly necessary.

- If any other institution or journal wishes to us to make our data available, this will only be done via a data-transfer agreement.

Specifically for focus group interviews:

- Transcripts of the interview will not become available
- Code trees will become available for research purposes.

Specifically for the MCD interventions and Train the Novice PhD-Supervisor

- The datafile with the training participants (anonymized) survey responses or (anonymized) suggestions provided to us will be available upon request.
- The themes of the dilemmas discussed in the moral case deliberation sessions.

**Data protection**

We use security and safety procedures against theft, loss and abuse by third parties and ensure that only authorized persons have access to the data (anonymized data and transcripts). Authorized persons are the members of the research team. The research team comprises the principal investigators ([names researchers]), potentially a masters intern of [researcher] and the statistician of the VU / VUmc.

**Data storage and location**

We will store your data for a maximum of fifteen years. Data for scientific research is stored according to the legal timeframe of data storage. The data is collected through [Qualtrics,](https://www.qualtrics.com/) which means that the data is stored on a European server. Data will be saved on Surfdrive servers. The audiotapes of the focus groups will be deleted and destroyed after we have transcribed the scripts verbatim. The experiences of participants with our interventions will be stored anonymously on Qualtrics.

**Dissemination of results for research**

We will analyze and publish the results of the survey for research purposes. Therefore, we will not include data on faculty, institution or university but will only stratify the data for disciplinary fields and academic ranks.

We will analyze and publish the results of the focus groups anonymously. It will be impossible to detect who participated in the focus groups from the transcripts/summaries of the focus groups.
We will analyze the results of the PhD training and Moral Case deliberation confidentially. If we are to publish the findings in scientific articles or educative website, we will do so anonymously.

**Dissemination of results for institutions**

The reports of the survey data for the four institutions will be aggregated at faculty level. Because universities are split into different faculties, they will receive reports on a faculty level. Medical centers are split into research institutes and will receive one report per institute. Summaries are only written if more than 25 active scientists filled in the survey and are working at the faculty. This will make it practically impossible to identify individual participants.

The reports of each focus group interviews will be sent to those participants to check if they have anything to add to the summary of the content of the focus groups. These reports will not be available for third parties nor the participating institutes.

The reports of the PhD supervisor training may be shared with the participating institutions for promotion purposes. The results of the Moral Case Deliberation sessions will not be shared, aside from participants’ experiences with the technique as well as global themes of the dilemmas.

**Questions**

If you have any questions, please send an email to [name researcher], department of philosophy via [email address project]. Your query will be dealt with as quickly as possible and will remain confidential.

**Withdrawing from the study**

If you would like to withdraw from the study, please send an email to [name researcher], department of philosophy via [[email address project]](mailto:info@amsterdamresearchclimate.nl%20) to discuss a possible withdrawal.
