# Supplementary material for: Researchers’ Perceptions of a Responsible Research Climate: A Multi Focus Group Study
Source: Sci Eng Ethics. 2020 Aug 10;26(6):3017–36. doi: 10.1007/s11948-020-00256-8 (PMC7755866; doi:10.1007/s11948-020-00256-8)
Supplement: Supplementary file 3 — 3. Informed consent form: Informed consent form for participation in the focus groups (DOCX 16 kb) [file 11948_2020_256_MOESM3_ESM.docx]

**Informed Consent Form**

This is the informed consent form for your participation in the focus groups organised for the Academic Research Climate in Amsterdam (ARCA) project.

**The study**

With this focus group, we aim to get a better picture of the research culture as experienced by you. On top of that, we want to discuss potentially effective interventions that could improve the research culture where necessary. The focus group takes on average no longer than 90 minutes.

**Confidentiality**

Everything you say will remain confidential. The transcribed interview, the analyses and final results will be formatted anonymously. If there are any questions or topics touched upon during the focus groups that you prefer not to answer, you should feel free not to do so.

**Questions**If you have any questions about the study, you can ask them prior to the focus groups. You can get in touch with me, [name researcher], via [email address researcher]. You can also reach project leader, [name project leader], directly via [email address researcher]

**Participant ’s consent**

- I have read the information letter. Also, I could ask questions. My questions have been answered adequately. I had enough time to decide whether I wanted to participate. I will treat the information from other participants in this focus group as confidential.

- I am aware that participation is voluntary. I also know that I decide to no longer participate in this focus group at any moment without having to provide a reason.

Name participant:

Signature: Date : XX/XX/XXX

-----------------------------------------------------------------------------------------------------------------

I declare that I have informed this participant satisfactorily about the above stated study.

If during the study any information is to become known that could affect the participant’s consent, I will inform him/her in time.

Name researcher (or researcher ’s representative)*:

Signature: Date: XX/XX/XXX

-----------------------------------------------------------------------------------------------------------------

* Strike out what does not apply.

*Participant receives a copy upon request*
