# Supplementary material for: Researchers’ Perceptions of a Responsible Research Climate: A Multi Focus Group Study
Source: Sci Eng Ethics. 2020 Aug 10;26(6):3017–36. doi: 10.1007/s11948-020-00256-8 (PMC7755866; doi:10.1007/s11948-020-00256-8)
Supplement: Supplementary file 4 — 4. Topic guide: Guide that addressed the questions posed in the focus groups (DOCX 19 kb) [file 11948_2020_256_MOESM4_ESM.docx]

**Introduction (10 min)**

- Thank the focus group participants for agreeing to the focus group
- Introduce focus group leader and observant as well as participants

- Check informed consent, if it is ok to record the interview, privacy policy and anonymity
- Explain what will happen with the results
- Explanation of how a focus group works, what are the rules for group discussions, etc.

- Ask them if they have any questions before the interview begins
- Brief introduction of this research project (refresh)

**Interview topics (3) to be covered during the focus group (80 min)**

1. Interactive assignment (think, pair, share). Assessment of the question: “What do you envision when thinking of a responsible research climate?” (25 min)
   1. Asks participants to individually write down three characteristics of responsible research climate (5 min). Participants are then invited to exchange these with their neighbours (5 min). Finally, groups are asked to share their characteristics with the group and discuss them deeper (10- 15 min)
2. Group discussion: “Which barriers do you perceive in the research climate for responsible conduct of research?” (20 min)

*I. for adequate mentoring?
 (can you give this to your mentees/are you provided this by your mentors/how does the
 hierarchical structure in your work play a role into this/how is role modelling relevant here?)
 (is the hierarchy in your department working in a way that it helps people in their research?)*

*II. for the most correct data analysis?*

*III. for optimal collaboration (inside and outside own department)?*

*IV. for carrying out your research faced with publication pressure or stress?*

*V. for complying with departmental rules of responsible research?
 VI. for adequate training and/or continues training?*

3b How would you tackle these barriers? (10 min)

*I (see above)*

*II*

*II etc.*

**Ending the interview (10 min)**

- Is there anything else you’d like to say?

- What is the main message for us to take away?

- Can I contact you in case I need any additional information of if something is unclear?

- What would you like to see coming out of our study?

- Close the interview and thank the interviewees for their participation.

- Report of this meeting with the option to comment and suggest corrections and mention the summary that will be sent to all participants to comment (member-check)
