# Supplementary figures and images for: Researchers’ Perceptions of a Responsible Research Climate: A Multi Focus Group Study
Source: Sci Eng Ethics. 2020 Aug 10;26(6):3017–36. doi: 10.1007/s11948-020-00256-8 (PMC7755866; doi:10.1007/s11948-020-00256-8)

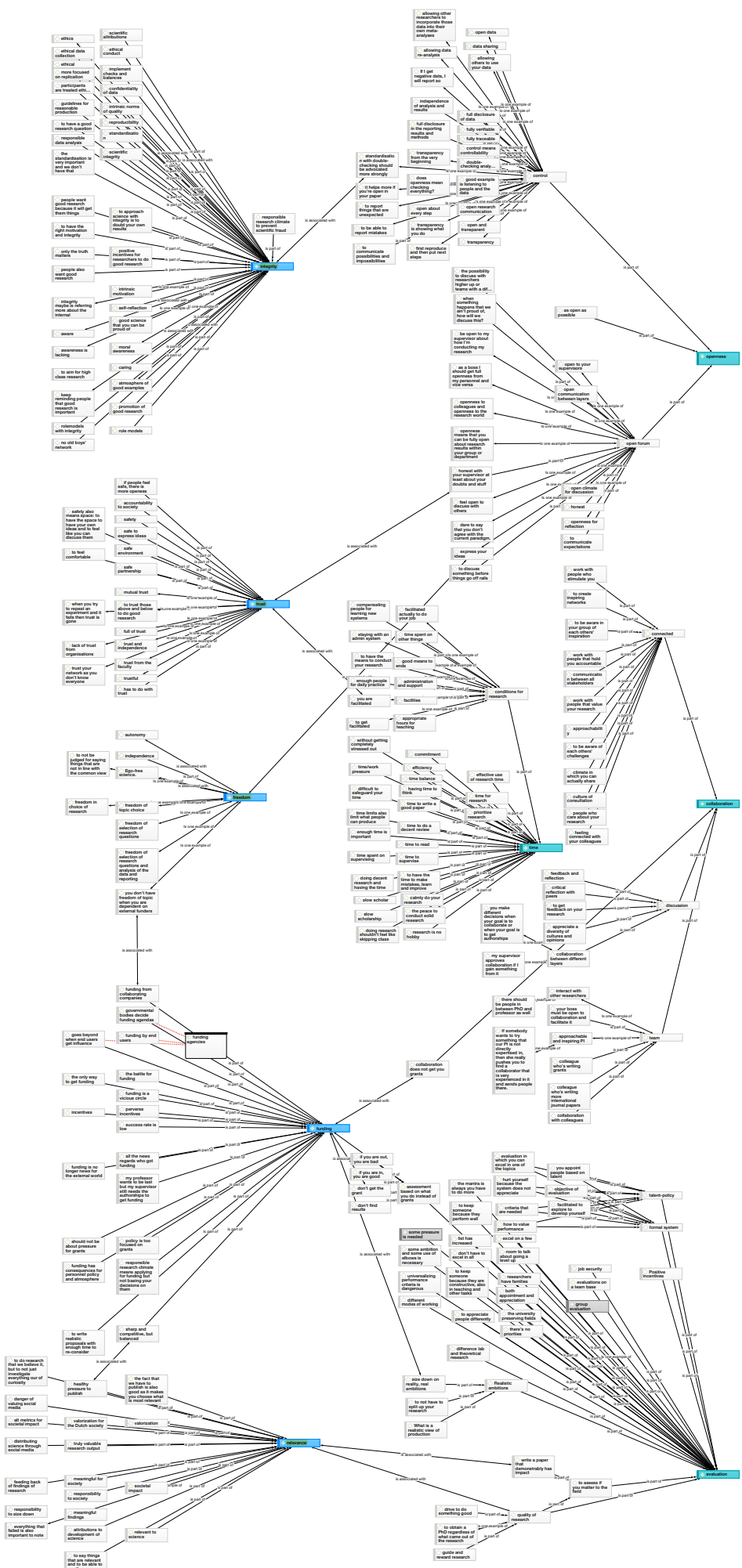

Supplement: Supplementary file 5 — 5. Coding scheme characteristics (PDF 233 kb) [file 11948_2020_256_MOESM5_ESM.pdf]
