# Supplementary material for: Researchers’ Perceptions of a Responsible Research Climate: A Multi Focus Group Study
Source: Sci Eng Ethics. 2020 Aug 10;26(6):3017–36. doi: 10.1007/s11948-020-00256-8 (PMC7755866; doi:10.1007/s11948-020-00256-8)
Supplement: Supplementary file 6 — 6. Coding scheme barriers and interventions (PDF 240 kb) [file 11948_2020_256_MOESM6_ESM.pdf]

- no money
- having to beg for money
- bad idea to learn something new
- lack of proper incentives
- cuts
- best to keep on doing the things I already did
- learning something new means publishing less
- pressure to publish
- chances of getting published vary

- funders determining the research agenda
- funding to work in an unsuccesful direction
- no trust in us that we do the right thing
- structurally too little funding
- not enough funding
- don't have funds
- funding for specific research

- heads of department that are not there as a choice

- the university wants higher research output
- impact fetish
- how do we evaluate
- don't get the opportunity
- lack time and still need to produce
- all time goes to producing own work
- lack of time
- little time to think
- getting the position without sufficient hours
- emphasis on quantity puts young moms at a disadvantage
- emphasis on quantity, not quality

- different ways of working
- no choice to develop how yo…
- to only note when someone underperforms
- to have to excel in everything
- now it's very individualistic
- you are doing alright
- individual evaluation
- publication requirements

- the six months leave to another university
- move from temporary contract to temporary contract
- no contract extension
- short term contract
- lack of continuity

- inconsequent technical support
- support staff has a greater role than the current budget allows
- there was no overlap
- not enough budget for support staff

- huge bureaucracy
- heavy institutionalising
- little time to read literature
- inflexible thinking about work dutie
- mentality of people who are in charge
- no autonomy
- the university wants more contact hours with students
- adjusting quality requirements
- administrative hassles
- administrative burden

- administrators that determine what your work space looks like
- architects that determine what workspace looks like
- little meeting places
- flexible working spaces
- Lacking the room
- no room for colloquia
- no place to have group meetings
- open-plan office
- no space to focus
- no place to concentrate
- unrestly process
- too little office spaces

- difficult organizing the time in teaching
- teaching is looked down upon
- teaching doesn't get rewarded

- the one who does only the teaching is never going to grow in his career
- teaching is pretty badly organized in general
- you always talk about teaching loads and research performance

- shift around the incentives
- allowing researchers to come up with their own topics
- to have academic freedom
- this pyramid had to change
- better governance of your PhD positions
- less PHD students and more post-docs
- acknowledge that there are more roads to funding
- less emphasis on many PhDs

- conduct science for society
- take a more trusting stand
- some stimulation to share science with society
- societal relevance
- not having to endlessly write proposals
- reaching out to the public
- less dependency on external funding
- value quality over H-index

- head expects us to be rolemodels
- head of department by choice
- assessment for group leaders

- greater focus on quality
- respecting our research time
- formal research time
- combine quantitative and qualitative assessment
- giving in to the fact that people have different preferences
- trust your staff to make promotion decisions
- everyone should get the chance to do research
- create research time for clinicians
- don't let grants determine personnel policy
- fair and diverse evaluati…
- make hiring decisions at the departmental or faculty level
- being a good employer saying, 'we care about you, we offer you a career path'…
- more objective set of criteria
- trust your professors
- non- monetary incentives
- grant people job security
- scientific excellence is broad

- good tenure track policy
- holistic policy
- to not be forced to score A's across the board
- to evaluate based on people's best output
- to take different disciplinary fields into account
- should be more specific to your field
- not treating everybody the same
- more relaxed and holistic view
- trust your staff to make hiring decisions
- take more time to hire people
- acknowledge public outreach in evaluation criteria
- acknowledge diversity in scientific excellence
- acknowledge differences between departments
- leave the focus on ranking behind

- to have continuity

- simplify organisational money structures
- to have a good library
- a stable basis of permanent staff
- support by people
- invest in support staff
- grants that take into account support staff
- assure good administrative staff
- good administrative support
- Realistic budgeting
- treat support staff as human beings
- benchfee for conferences and ECTs

- make the internal requests easy
- to get rid of administrative hassles
- value quality over quantity
- diminish the administrative hassle
- abolish some of the ridiculous administrative requirements
- more respect for the scientists
- decent research infrastructure
- empirically supported policies
- propose measures that you can provide the infrastructure for
- better infrastructure
- admin-free time
- Put a good financial system in place
- time to focus on a good paper

- to be facilitated in going to relevant conferences
- support from your department
- get rid of the flexible work spaces

- learn researchers how to teach
- teaching has to get rewarded on a group level
- teaching should be better appreciated
- value good teaching evals
- assign research blocks
- to get time to do research whilst specialising

- teaching-free time
- time divided half-half, half teaching, half research
- research time compartments
- invest in teaching quality management and statistics
- to give clinicians time to do research

- I once said to my supervisor that I have pain in my arms and neck from working. She said, yeah, I've had that for twenty years already so…

- culture with not too many compliments
- normalizing over hours
- feeling pressured to produce
- you're not a good researcher if you're not working in the weekends
- time pressure
- not enough time
- normal to work 60-70 hours a week
- expected to do research in own time
- it's good to say that you're working on the weekends
- feeling you cannot make mistakes

- norm is that you are available full time
- don't give to many A's
- they appreciate me for responding to them on the weekend
- to feel stressed when you are not 24/…
- work is never finished
- there is less peer review and you have to write more
- you do not want a burnout
- people never stop working
- getting the job and not daring to complain
- not helping

- colleagues who are very conscious about when they are sending emails
- less research but qualitatively better research
- have that one week for myself
- have a house near the seaside
- giving compliments
- celebrate successes
- everybody makes mistakes
- appreciate your researchers
- stick to office hours and not go over time
- university offering sports
- sport for free
- autonomy or independence in the way you organize your work
- develop a pride in the fact that we have research time
- they make sure not to send their PhDs replies on weekends or in the evenings because they don't want to get that message across

- motivate your researchers to do even better
- communicating the amount of time you actually work
- supervisors gave me the advice to really work in the office and not work at home
- create breaks
- Set limits, say no, whatever
- don't feel like a bad colleague if you don't reply immediately
- don't expect response within the weekend
- a head of department that sets the good example
- healthy to do something else than work.
- sometimes need to take a break as well
- I see it as an experiment, how long do I dare to not answer this e-mail?

- unconscious rolemodelling
- supervisors don't validate you
- no expert but doing the work
- no experience with other research groups
- giving a PhD candidate 3 years but demanding the quality of 4
- have to publish to graduate
- courses that are too general
- money should be no barrier to acquire the relevant research skills
- we have to the highest academic degree, yet we struggle hire to find a job
- to be trained in one area only
- people have to finish their dissertation in their own time
- a lot of PhD hired and there's really no care about their life after
- PhD candidates needing extensi…
- PhDs that are presumably educated to be helpful outside academia
- odds of a succesfull career are low

- start from scratch to train themselves
- inflexible supervisors
- pyramid of PhDs
- PhDs that are cheap
- Differences in expertise between your supervisors
- you're doing a PhD, it's part of the job to work seven days a week
- PI's that don't care about science

- reconsider requirements for PhD thesis
- loosen the demand to publish during PhD
- assess PhD based on ability not publications
- adjust the PhD thesis requirements
- assuring PhDs can defend on time

- cultivate assertiveness in PhDs
- have time to foster development in PhD students
- strong characters
- a course on how to publish your first paper
- to give PhD students realistic teaching loads
- time to debate with your supervisor
- investing in soft skills and personal development
- getting credits for personal development
- clarity about what kind of courses are available
- choosing a research profile
- to assure statistical education for PhD students
- encourage skepticism

- to have a mentor
- give all PhDs a daily supervisor
- PhDs that get a promotor and a daily supervisor
- to have a daily supervisor
- to stimulate young people under his charge and leadership
- supervisor that can give you confidence
- provide a learning environment to sit down with PhD students
- make supervision training compulsory
- develop a mentor system
- intervision for supervisors
- intervision
- formalising what is expected of a supervisor
- stable supervision meeting times
- supervision course
- supervisor course
- supervising for supervisors
- supervision manual
- research integrity course for supervisors
- supervisors setting the example for responsible research

- you have all these things which for society and science as a whole would be better to do A and the incentive is always to do B
- we sacrifice careful research for the X factor
- need to recycle your own thoughts all the…
- torture the data until something com…
- to go fast instead of checking everything
- no proper training
- no room for exploration

- start replicating ever…
- research integrity course
- time to discuss research ethics
- method to structurally discuss RI
- discuss research integrity again later
- just because someone publishes something doesn't mean it…
- integrity check-in
- group meeting to discuss research integrity
- follow up research integrity course
- every PhD should start the replication project
- code and data should be completely open
- if you hire someone, you must guarantee the conditions for responsible research
- be less trusti…
- appoint a confidential advisor
- be honest about sloppiness
- seminar on online data management
- meeting with your promoter and co-promoter to discuss research integrity
- don't let people get away with substandard research
- take time to replicate our studies
- integrity debates on research days
- teach a variety of analysis techniques
- compulsory research integrity training across the organisation
- to share best practices
- data storage facilitation
- protect your whistle blowers
- to have a good research question

- people may steal work
- no experience in your group
- nepotism
- political game
- polical intricacies
- secrecy
- digging your own grave by not sharing
- no collaboration

- lack of exchange between different disciplines
- lack of exchange between different departments
- bad communication

- to discuss things when they are at issue
- to be able to discuss things openly
- to work on the confidence to discuss your flaws
- room for discussion
- balance on the one hand between being honest about things that happened, things that should be different and people being willing to hear what someone else has to say…
- know what other people doing to appreciate them
- team evaluation
- make room for collaboration in evaluation criteria
- stop the press around who got funding
- group evaluation
- Get experience elsewhere

- website with all groups and their specialisat…
- website with all groups and the techniques they use
- website where all research projects that overlap are made clear
- finding a balance between honest and harsh
- more collaboration
- reward collaboration
- acknowledge contribution to larger research projects
- keep be curious to learn about people- irrespective of their rank
- interaction among colleagues
- team products
- assure to include everyone that contributed
- share your experimental c…
- count your codes/data as 'product'
